# Urinary angiotensin-converting enzyme 2 and its activity in cats with chronic kidney disease

**DOI:** 10.3389/fvets.2024.1362379

**Published:** 2024-05-02

**Authors:** Tzu-Chien Kuo, Wei-Li Hsu, Vin-Cent Wu, Tong-Rong Jan, Pei-Shiue Jason Tsai, Ya-Jane Lee

**Affiliations:** ^1^Institute of Veterinary Clinical Science, School of Veterinary Medicine, College of Bio-Resources and Agriculture, National Taiwan University, Taipei, Taiwan; ^2^Graduate Institute of Microbiology and Public Health, College of Veterinary Medicine, National Chung Hsing University, Taichung, Taiwan; ^3^Department of Internal Medicine, National Taiwan University Hospital, Taipei, Taiwan; ^4^Department of Veterinary Medicine, School of Veterinary Medicine, National Taiwan University, Taipei, Taiwan; ^5^National Taiwan University Veterinary Hospital, College of Bio-Resources and Agriculture, National Taiwan University, Taipei, Taiwan

**Keywords:** ACE2, urinary ACE2, RAAS, CKD, feline, small animals

## Abstract

**Introduction:**

Angiotensin-converting enzyme 2 (ACE2) played an important role in the renin-angiotensin-aldosterone system (RAAS) and it was proved to be renoprotective in renal disease. Urinary angiotensin-converting enzyme 2 (uACE2) has been shown to reflect renal injury in human and experimental studies, but its role in feline kidney disease remains unknown.

**Aims:**

Our objectives involve comparing uACE2 concentrations and activities in cats across CKD stages with healthy controls, investigating the relationship between uACE2 concentrations, activities, and clinicopathological data in feline CKD patients, and assessing the predictive abilities of both for CKD progression.

**Methods:**

A retrospective, case–control study. The concentration and activity of uACE2 were measured by commercial ELISA and fluorometric assay kits, respectively. The concentration was adjusted to give uACE2 concentration-to-creatinine ratios (UACCRs).

**Results:**

In total, 67 cats consisting of 24 control and 43 chronic kidney disease (CKD), including 24 early-stage CKD and 19 late-stage CKD, were enrolled in this study. UACCR values were significantly higher in both early-stage (2.100 [1.142–4.242] x 10^−6^) and late-stage feline CKD (4.343 [2.992–5.0.71] x 10^−6^) compared to healthy controls (0.894 [0.610–1.076] x 10^−6^; *p* < 0.001), and there was also significant difference between-early stage group and late-stage group (*p =* 0.026). Urinary ACE2 activity (UAA) was significantly lower in CKD cats (1.338 [0.644–2.755] x pmol/min/ml) compared to the healthy cats (7.989 [3.711–15.903] x pmol/min/ml; *p* < 0.001). UACCR demonstrated an independent, positive correlation with BUN (*p* < 0.001), and UAA exhibited an independent, negative correlation with plasma creatinine (*p* < 0.001). Both UACCR and UAA did not yield significant results in predicting CKD progression based on the ROC curve analysis.

**Conclusion and clinical importance:**

uACE2 concentration and activity exhibit varying changes as renal function declines, particularly in advanced CKD cats.

## 1 Introduction

The renin-angiotensin-aldosterone system (RAAS) is influential in chronic kidney disease (CKD) pathophysiology, and RAAS activation is a crucial factor in CKD progression in humans (1). RAAS is thought to be a linear and maladaptive system that activates the angiotensin-converting enzyme (ACE)-angiotensin II (Ang II)-angiotensin II type I receptor (AT1R) axis (2). In addition to inducing potent vasoconstriction, conserving sodium and water, and releasing aldosterone, this axis also has pro-inflammatory, pro-oxidative, and pro-fibrosis effects (3). Treatment with ACE inhibitors (ACEi) and angiotensin receptor blockers (ARB) to control RAAS for proteinuria and/or hypertension is widely accepted as the first-line therapy for CKD in human/veterinary medicine (4–6).

Angiotensin-converting enzyme 2 (ACE2), a homolog of ACE, was discovered in 2000 (7). Similar to ACE, ACE2 is a membrane-bound zinc metalloproteinase expressed in various organs such as the cardiovascular system and kidneys (7). However, unlike ACE, ACE2 is a mono-carboxypeptidase and has a catalytic ectodomain that generates angiotensin (1–7) directly from Ang II (8) angiotensin (1–7) mainly binds to the endogenous Mas receptor (MasR) and thus the biological effects related to angiotensin (1–7), namely vasodilation, anti-oxidation, anti-inflammation, and anti-fibrosis, are opposite to those of Ang II in humans and experimental animals (9). In cats, combining ARB plasma with recombinant human ACE2 also results in an increase in angiotensin (1–7) (10). This suggests that the ACE2-angiotensin (1–7)-MasR axis is a counterbalance to the ACE-Ang II-AT1R axis during RAAS (11). Furthermore, a decrease in renal ACE2 expression has been reported to be associated with renal injury and related complications, including albuminuria in kidney injury animal models (12, 13). Thus, it is likely that ACE2 plays a protective role in kidney disease (14, 15).

The ectodomain of ACE2 can be cleaved by enzymes, including disintegrin and metalloproteinase-17 (ADAM-17) (16, 17), and then the protein ultimately is shed into urine as urinary ACE2 (uACE2). Research indicates that renal tubular ACE2 levels in diabetic kidney disease mice correlate with uACE2 levels but not with plasma or serum ACE2 concentration (18). Moreover, injecting recombinant ACE2 markedly increase serum ACE2 but not urinary ACE2 (19), uACE2 reflects ACE2 release from proximal tubules (20, 21) and increases in both humans and mice with CKD and diabetic kidney disease (DKD) (21, 22). In addition, albuminuria and elevated biomarkers related to tubulointerstitial injury have been associated with increased uACE2 in individuals with CKD (23).

CKD commonly affects cats, especially the elder ones (24). Specifically, the histopathological expression of renal ACE2 and its relationship with renal damage have been described in CKD cats (25). However, to the best of our knowledge, the role of uACE2 in CKD cats remains unknown. Thus, our aims are several folds, including: (1) to carry out a comparative analysis against healthy controls of uACE2 concentrations and activities in cats across the different stages of CKD; (2) to investigate the relationship between uACE2 concentrations, activities, and clinicopathological information obtained from feline CKD patients; (3) to evaluate the predictive ability of uACE2 concentrations and activities in relation to CKD progression.

## 2 Materials and methods

### 2.1 Patients and sample collection

The urine samples were collected from client-own cats that attended National Taiwan University Veterinary Hospital from September 2019 to March 2022, and all the urine samples were aliquoted into 1.5-ml Eppendorf tubes, and stored in a − 80°C deep freezer until further analysis. A detailed description of each case was recorded for identification purposes, including distinctive features and, clinical information, including history, physical examination results, and hematology results such as plasma biochemistry, plasma electrolyte concentrations, and urinary analysis. Any cases diagnosed with neoplasm, an endocrine disease (e.g., hyperthyroidism, hypothyroidism, diabetes mellitus, hyperadrenocorticism or hypoadrenocorticism), heart disease (e.g., hypertrophic cardiomyopathy or, chronic degenerative valve disease), a systemic infectious disease (e.g., blood parasites, sepsis or viral diseases such as feline infectious peritonitis or feline leukemia virus), a urinary tract infection/obstruction, a confirmed gastrointestinal disease, or a diagnosed liver disorder were excluded from this study. In addition, any cases prescribed with angiotensin-converting enzyme inhibitor (ACEi) and angiotensin receptor blocker (ARB) were also excluded.

Based on the above information, the sample population was classified into the following groups. The control group consisted of cats without any clinical signs, who were not receiving any medication, and had unremarkable physical examination findings, as well as had normal values for blood urea nitrogen (BUN; 16–36 mg/dL), plasma creatinine (0.8–1.8 mg/dL), and symmetric dimethylarginine (SDMA; <14 μg/dL). All cases selected into the control group also had normal CBC values, a normal albumin, urine specific gravity (USG) >1.035, urine protein-to-creatinine ratio (UPC) < 0.2 and without active sediments in the urine. While CKD group retrospectively selected feline cases which met at least two of the following appropriate criteria over at least 3 months. These included having clinical signs (e.g., polyuria), azotemia (creatinine >1.6 mg/dL), an abnormal urinalysis (e.g., USG < 1.030 without identifiable non-renal cause, or persistent proteinuria, which was defined as UPC > 0.4 on three occasions and more than 2 weeks apart) or abnormal renal imaging findings consistent with CKD, such as small irregular kidneys, renal infarct, cyst, or decreased corticomedullary distinction.

All CKD cats were then classified into four stages based on the International Renal Interest Society (IRIS) CKD staging system. Owing to the small number of CKD stage 1 and stage 4 cases, these CKD groups were alternatively divided into IRIS early stage and IRIS late stage composed of patients at IRIS stage 1 and stage 2 CKD, or patients with IRIS stage 3 and 4, respectively. The progression was defined as an increment in plasma creatinine concentration > 0.5 mg/dL within a follow-up period of 6 months for CKD cats, excluding cases with documented dehydration from medical records.

### 2.2 Urinary ACE2 concentration

Feline uACE2 concentrations were measured by cat-specific commercial quantitative sandwich ELISA kit (Cat. No: MBS085876, Mybiosurce, United States), following the procedure described in the manufacturer’s instruction manual. The standard curve was established using standard samples at concentrations of 0.25, 0.5, 1.0, 2.0, 4.0, and 8.0 (ng/ml), which were supplied in the kit. The sensitivity of these kits is 0.1 (ng/ml) and the detection range of these kits is 0.25–8.00 (ng/ml). Each urine sample was measured in duplicate and the final concentration was expressed as the mean of the two concentrations. The intra-assay CV (%) and inter-assay CV (%) were < 5% and < 8% when using these kits, respectively (Supplementary Table S1).

### 2.3 Urinary ACE2 activity

Feline uACE2 activity (UAA) was measured using an methoxycoumarin (MCA)-based peptide substrate in a commercial ACE2 activity assay kit (ab273297, Abcam, United Kingdom) according to the manufacturer’s instructions with modifications based on a previous method for a urinary sample (18, 26). This kit utilizes the ability of an active ACE2 to cleave a synthetic MCA-based peptide substrate to release a free fluorophore. The released MCA can be quantified using a fluorescence microplate reader (SpectraMax^®^ M5, Molecular Devices LLC, US) that measures the sample’s relative fluorescence units (RFU) with an excitation (Ex) wavelength of 320 (nm) and an emission (Em) wavelength of 420 (nm) using 96-well black plates (No:655076, Greiner Bio-One, Thailand). According to the instruction manual, the lower limit of the ACE2 fluorometric assay kit is 0.4 pmol/min. Based on the MCA-standard curve, the available detection range of ΔRFU is 60 to 360.

Briefly, the assay procedure used was as follows. The MCA-standard curve was established by following the manufacturer’s instructions. The fluorescence (Ex/Em = 320/420 nm) of each MCA standard was measured in duplicate and each final fluorescence was expressed as the mean of the two values in an end mode. Subsequently, 2 microliters (μl) of urine sample (S) with or without being diluted 1:1 with distilled water was added to plate wells in triplicate. Next, 2 μL of the prepared ACE2 inhibitor was added to one of the wells containing the sample to serve as a negative control (NC). The volume of the S and NC samples was then adjusted to 50 μL per well using ACE2 assay buffer, and incubated for 15 min (min) at RT. After incubation, 50 μL ACE2 substrate mix was loaded into the S and NC wells. Measurement of fluorescence (Ex/Em = 320/420 nm) tool place every 8 min in kinetic mode for 64 min. If the R^2^ of each sample is >0.95, any two-time points (T_1_ and T_2_) in the linear range of the plot were selected and the corresponding values for the fluorescence (RFU_1_ and RFU_2_) were estimated; following this, the ACE2 activity of the sample was calculated using the formula in the product instruction manual.

### 2.4 Statistical analysis

Statistical analysis of the study population was performed using SPSS software (v.25). Non-parametric statistics were employed due to the insufficient sample size to achieve a normal distribution. The continuous data are presented as medians (interquartile range) based on the Shapiro–Wilk test (n≦50). The Mann–Whitney U test was used to compare between two groups, and the Kruskal-Wallis test was used to compare between more than two groups; this was followed by Dunn’s multiple comparison tests. The Chi-square test or Fisher’s exact test was used for analyzing differences between categorical variables. A significant difference was defined as *p* < 0.05. Univariable and stepwise multivariable linear regression analyses were used to assess the association between uACE2 and various variables. Receiver operator characteristic (ROC) curve analysis was performed to predict renal progression and to determine the best cut-off values.

## 3 Results

### 3.1 Patient s’ grouping

This study collected urine samples from 67 cats, including 24 healthy cats and 43 cats with CKD. These consisted of 29 domestic shorthairs, 2 American Shorthair cats, 1 Exotic shorthair, 3 Scottish folds, 5 Persians, 2 British longhairs, 2 Ragdolls, 1 Russian blue, and 22 cats with unrecorded breeds. Based on the IRIS CKD staging system, all cats with CKD were subclassified into CKD stage 1 (*n* = 1), CKD stage 2 (*n* = 23), CKD stage 3 (*n* = 17), and CKD stage 4 (*n* = 2). Due to only 1 cat with CKD stage 1 and 2 cats with CKD stage 4, all CKD cats were then classified into two groups, i.e., an early-stage group and a late-stage group. The early-stage group (*n* = 24) consisted of cats with CKD stage 1 and CKD stage 2, whereas the late-stage group (*n* = 19) consisted of cats with CKD stage 3 and CKD stage 4.

### 3.2 Urinary angiotensin-converting enzyme 2 (uACE2) among the control animals and the animals with CKD and its different stages

**Figure 1:**
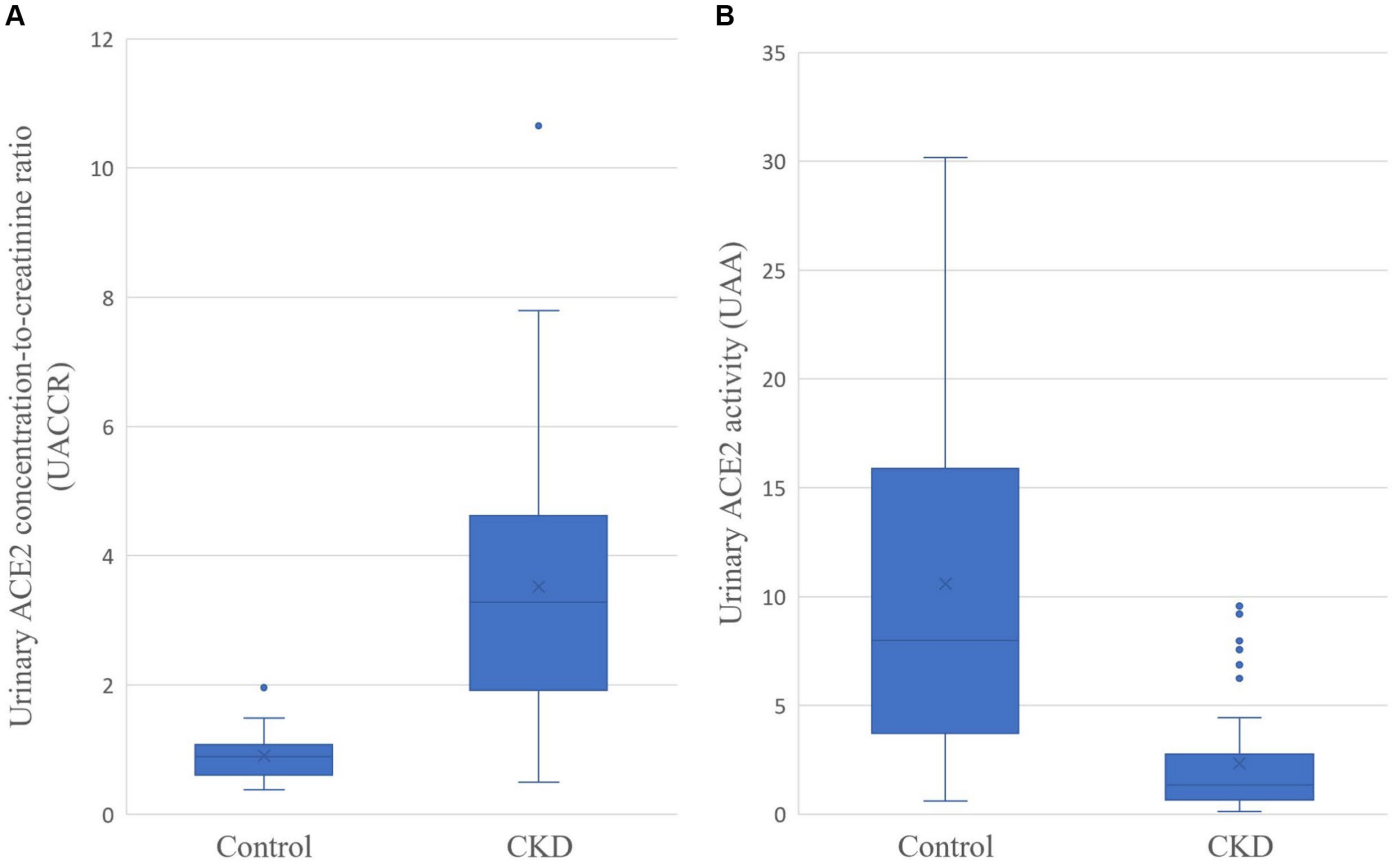
Urinary ACE2 concentration-to-creatinine ratio (UACCR; x 10^−6^; **A**) and urinary ACE2 activity (UAA; pmol/min/ml; **B**) in control and CKD cats. The *p*-values for both groups are less than 0.001.

Figure 1 revealed the difference in urinary ACE2 concentration-to-creatinine ratio (UACCR) and urinary ACE2 activity (UAA) between the feline control group and the CKD group. The CKD cats had significantly higher UACCR (3.284 [1.918–4.616] x 10^−6^; *p* < 0.001) but lower UAA (1.338 [0.644–2.755] x pmol/min/ml; *p* < 0.001) compared to healthy cats (UACCR:0.894 [0.610–1.076] x 10^−6^; UAA: 7.989 [3.711–15.903] x pmol/min/ml).

Table 1 summarizes data on feline UACCR levels in cats with early-stage CKD (2.100 [1.142–4.242] x 10–6) and late-stage CKD (4.343 [2.992–5.0.71] x 10–6), indicating higher values compared to healthy cats (0.894 [0.610–1.076] x 10–6; *p* < 0.001). Late-stage CKD cats also showed higher UACCR than early-stage CKD cats (*p* = 0.026). Early-stage (1.482 [0.801–4.150] pmol/min/ml) and late-stage (0.984 [0.546–2.056] pmol/min/ml) CKD groups exhibited significantly lower UAA values than healthy cats (7.989 [3.711–15.903] pmol/min/ml; *p* < 0.001), with a decreasing trend from early to late-stage CKD. Cats with late-stage CKD had higher plasma creatinine (*p* < 0.001) and BUN (*p* < 0.001) compared to healthy and early-stage CKD cats. Similar trends were observed for UPC (*p* < 0.001 and *p* = 0.004 compared to healthy and early-stage CKD, respectively). Significantly lower USG values were noted in late-stage CKD compared to healthy and early-stage CKD cats (*p* < 0.001 and *p* = 0.015, respectively). Importantly, BUN and UPC showed no significant differences between the healthy and early-stage CKD groups.

**Table 1:** Clinical characteristics of cats classified into the control group, the CKD early stage group, and the CKD late-stage group.

	CKD groups					
Parameters	Control	*n*	Early stage	*n*	Late stage	*n*
UAC (ng/ml)	3.279 (2.813–3.748)	24	3.436 (3.096–3.622)	24	3.543 (3.197–3.794)	19
UACCR (x 10^−6^)	0.894 (0.610–1.076)	24	2.100 (1.142–4.242)^a^	24	4.343 (2.992–5.0.71)^a,b^	19
UAA (pmol/min/ml)	7.989 (3.711–15.903)	24	1.482 (0.801–4.150)^a^	24	0.984 (0.546–2.056)^a^	19
Gender (male %)	50.0	24	62.5	24	63.2	19
Age (year)	6.5 (2.5–8.0)	24	7.0 (5.0–10.0)	23	13 (7.0–15.0)^a,b^	19
Creatinine (mg/dL)	1.5 (1.2–1.6)	24	2.1 (1.8–2.5)^a^	24	3.4 (3.0–4.4)^a,b^	19
BUN (mg/dL)	21 (19–25)	24	23 (21–28)	24	40 (35–53)^a,b^	19
SDMA (μg/dL)	9.0 (6.5–11.0)	24	10 (8–17)	6	20 (9–30)	4
Hematocrit (%)	41.2 (36.4–45.3)	24	42.3 (38.7–45.3)	24	37.0 (34.5–40.1)	19
RBC (10^6^/μL)	9.05 (8.28–9.65)	24	9.08 (8.12–9.74)	24	8.09 (7.37–8.76)	19
Hemoglobin (g/L)	14.4 (12.5–15.8)	24	14.3 (13.2–15.6)	24	12.7 (12.2–13.9)	19
WBC (/μL)	7,200 (5550–9,100)	24	6,200 (5375–8,675)	24	8,600 (5500–11,100)	19
PLT (K/μL)	256 (134–308)	24	275 (224–457)	24	300 (203–412)	19
Albumin (g/dL)	3.6 (3.3–3.8)	24	3.5 (3.3–3.7)	19	3.5 (3.2–3.7)	15
ALKP (U/L)	37 (33–45)	20	36 (32–57)	14	34 (27–41)	9
ALT (U/L)	52 (44–67)	20	53 (36–61)	14	68 (48–97)	9
AST (U/L)	38 (28–46)	20	29 (24–48)	14	34 (27–45)	9
Glucose	115 (101–150)	24	110 (98–131)	16	109 (99–130)	10
Phosphate (mg/dL)	-----	0	4.2 (3.4–4.6)	20	4.5 (3.7–5.0)	18
Sodium (mEq/L)	155.6 (154.1–157.8)	18	154.9 (153.6–156.6)	21	154.6 (152.5–158.1)	19
Potassium (mEq/L)	3.72 (3.48–3.90)	18	3.95 (3.46–4.12)	21	3.96 (3.68–4.41)	19
Chloride (mEq/L)	117.4 (114.5–119.4)	18	117.4 (115.7–119.6)	21	117.0 (115.7–118.7)	19
Urinary SG	1.049 (1.045–1.055)	24	1.017 (1.013–1.040)^a^	24	1.011 (1.009–1.014)^a,b^	19
Urinary pH	6.26 (6.06–6.53)	24	6.11 (5.80–6.56)	24	6.01 (5.69–6.40)	19
UPC	0.03 (0.03–0.04)	24	0.06 (0.02–0.15)	24	0.19 (0.10–0.29)^a,b^	19

^a^ and ^b^ indicate significance relative to the control and early-stage groups, respectively. The post hoc analysis was Dunn’s test. ACE2, angiotensin-converting enzyme 2; ALT, alanine aminotransferase; ALKP, alkaline phosphatase; AST, aspartate aminotransferase; BUN, blood urea nitrogen; PLT, platelet count; RBC, red blood cell; SDMA, symmetric dimethylarginine; UAA, urinary ACE2 activity; UAC, urinary ACE2 concentration; UACCR, urinary ACE2 concentration-to-creatinine ratio; UPC, urine protein-to-creatinine ratio; Urinary SG, urinary specific gravity; WBC, white blood cell.

### 3.3 Correlation between urinary angiotensin-converting enzyme 2 (uACE2), urinary ACE2 activity (UAA), and other clinicopathological parameters

The results of the univariable and stepwise multivariable linear regressions for the various factors associated with UACCR and UAA in cats are summarized in Table 2. UACCR was positively associated with age (*p* = 0.002), plasma creatinine (*p* < 0.001), BUN (*p* < 0.001), and UPC (*p* < 0.001), while being negatively related with HCT (*p* = 0.016), ALB (*p* = 0.037), USG (*p* < 0.001), urinary pH (*p =* 0.019) and UAA (*p =* 0.002) when using univariable linear regression; In univariable linear regression, USG (*p* < 0.001) and urine pH (*p =* 0.004) were significantly positive associated factor with UAA, then plasma creatinine (*p* < 0.001), BUN (*p* < 0.001), and UAACR (*p =* 0.002) were negative correlation with UAA. Moreover, using stepwise multivariate linear regression analysis (Table 2), BUN (*p* < 0.001) and USG (*p* < 0.001) were independent factors associated with UACCR (R^2^ = 0.806), while plasma creatinine (*p* < 0.001) and urine pH (*p =* 0.045) were independent factor for UAA (R^2^ = 0.262).

**Table 2:** Univariable and stepwise multivariable linear regression analyses for factors associated with UACLR and UAA in cats.

	UACCR					UAA				
	Univariable		Multivariable (R^2^ = 0.806)			Univariable		Multivariable (R^2^ = 0.262)		
Parameters*	Adjusted *β*	*p*	Adjusted *β*	95% CI	*p*	Adjusted *β*	*p*	Adjusted *β*	95% CI	*p*
Age	0.379	0.002	-----	-----	-----	−0.151	0.228	-----	-----	-----
SAP	0.187	0.382	-----	-----	-----	0.244	0.251	-----	-----	-----
HCT	−0.294	0.016	-----	-----	-----	0.199	0.107	-----	-----	-----
ALB	−0.274	0.037	-----	-----	-----	0.045	0.738	-----	-----	-----
pCrea	0.652	< 0.001	-----	-----	-----	−0.487	< 0.001	−0.423	−4.325 - -1.358	< 0.001
BUN	0.792	< 0.001	0.679	0.067–0.115	< 0.001	−0.340	0.005	-----	-----	-----
phosphate	0.307	0.051	-----	-----	-----	−0.121	0.451	-----	-----	-----
USG	−0.730	< 0.001	−0.365	−0.084 - -0.028	< 0.001	0.461	< 0.001	-----	-----	-----
Urine pH	−0.285	0.019	-----	-----	-----	0.347	0.004	0.225	0.061–5.720	0.045
UPC	0.604	< 0.001	-----	-----	-----	−0.229	0.063	-----	-----	-----
UACCR						−0.372	0.002			
UAA	−0.375	0.002	-----	-----	-----	-----	-----	-----	-----	-----

*The parameters were listed and selected when their association was potentially significant (*p* < 0.1) for either UALCR or UAACR. ALB, albumin; BUN, blood urea nitrogen; HCT, hematocrit; RBC, red blood cell; pCrea, plasma creatinine; SAP, systolic arterial pressure; UAA, urinary ACE2 activity; UACCR, urinary ACE2 concentration-to-creatinine ratio; USG, urinary specific gravity; UPC, urine protein-to-creatinine ratio.

### 3.4 Assessment of the predictive ability of UACCR and UAA in relation to renal progression

We attempted to determine whether UACCR or UAA could serve as a predictor for feline CKD progression, which was defined as an increment in plasma creatinine concentration > 0.5 mg/dL within a follow-up period of 6 months for CKD cats. Only 26 cats can get the progression data during this six-month follow-up period, and 7 achieved progress. However, neither of these factors demonstrated a significant difference when using ROC curve analysis (*p =* 0.370 for UACCR and *p =* 0.140 for UAA). The area under the ROC curve (AUC) of UACCR and UAA was 0.617 and 0.692, respectively (Supplementary Figure 1).

## 4 Discussion

Although the relationship between uACE2 concentration and activity in the context of renal disease has been reported for human medicine and using experimental models, to the best of our knowledge this is the first study to simultaneously measure the concentration and activity of uACE2 in cats. Moreover, a comparison of these two measurements between CKD subjects and healthy subjects and among CKD subgroups was carried out. This was expanded into an investigation of their correlation with clinicopathological features. In the present study, UACCR was significantly higher in CKD cats compared to healthy controls. This result is similar to a previous study investigating urinary ACE2 concentration in humans (21). Additionally, the higher UACCR with advanced CKD was consistent with human findings, and, specifically, a decline in renal tubular function has previously been recognized as an important factor that is associated with increased ACE2 shedding (23).

In a previous study in humans and mice with chronic renal disease, both uACE2 protein concentration and activity were consistently increased (22), which implies that most of the uACE2 protein retains enzymatic activity and that total UAA increases as more ACE2 protein is shed into the urine. However, in the current study, it was observed that cats afflicted with CKD exhibited a notably reduced UAA when compared to healthy controls. This finding stands different from the results obtained from mice and humans (22). Interestingly, a previous study found that shed ACE2 may be cleaved, and ultimately yielded two different molecular forms of ACE2 in urine (27). These researchers further suggested that species-specific cleavage sites may exist (18, 28). Consistently, another study has also revealed that different molecular forms of the protein exist in healthy humans and CKD patients (21). The presence of different cleavage sites not only results in different molecular forms of ACE2 in urine but also implies that these different molecular forms may have different activities. It is important to note that Arg^577^ and Lys^596^ have been identified as the cleavage sites that produce shorter ACE2 fragments in mouse proximal tubular cells (29), and these amino acids are also conserved in the human (NCBI sequence ID: NP_001358344.1) and canine ACE2 (NCBI sequence ID: NP_001158732.1); however, Arg^577^ is replaced with Thr^577^ in feline ACE2 (NCBI sequence ID: XP_023104564.1). Therefore, it is possible that there are different molecular forms of uACE2 present in healthy cats and in CKD cats, and that the modified uACE2 may lose its activity after being cleaved previously in CKD cats. Further studies are needed to confirm the presence of these different ACE2 molecular forms in cats and to measure separately the activity of the various molecular forms of ACE2 in CKD and healthy feline urine.

In the present study, both UACCR and UAA demonstrated independent associations with renal indexes, with UACCR exhibiting a positive correlation with BUN and UAA showing a significant but weak negative correlation with plasma creatinine. The findings of UACCR align with previous human studies (23), though there was no analogous study regarding UAA. As above, the variability of UAA in cats differs from that observed in humans and experimental animals, Therefore, investigation into the role of UAA in cats with CKD is warranted for future research endeavors.

USG, indicative of urine concentration ability and a component of renal function, is also affected by the size of molecules within urine (30), it was found to be significantly, independently, and negatively correlated with UACCR in cats in the present study. Both an increase in uACE2 and a decrease in renal ACE2 have been reported to be associated with tubular injury in renal disease, which in turn may affect renal function (23, 31). The mechanism linking USG and ACE2 remains unclear and requires further investigation through additional studies.

Our findings show that both UACCR and UAA in cats are not significantly associated with UPC. Indeed, previous investigations exploring the histopathological change in renal injury in cats have indicated that renal injury tends to more prominent affect the tubulointerstitium but not the glomerulus (32). Furthermore, A recent study revealed no association between renal ACE2 expression and renal damage in feline CKD based on histopathology (25). In small animal clinical practice, persistent renal proteinuria usually is due to glomerular injury when dogs have renal disease, whereas this seldom occurs in cats with renal disease (33). However, to eliminate the impact of ACEI/ARB, we also excluded cases with proteinuria, as they can be prescribed with ACEI/ARB, from our study. While this refinement ensures that the results specifically apply to cats with lower urinary protein levels, it may definitely underpower the association between UACCR, UAA, and UPC.

In our study, a positive correlation was found between urinary pH and UAA in cats with CKD. Previous research suggests that ACE2 substrate hydrolysis rates are pH-dependent, but the relationship between urinary ACE2 activity and pH may not be linear (34). Additionally, species-specific variations in the effect of pH on urinary ACE2 activity were noted (34). However, further investigation is required to elucidate the actual mechanism by which pH affects UAA alterations.

There were several limitations to our study. Firstly, we have a relatively small sample size for cats (*n* = 67). These smaller sample sizes may decrease statistical power and affect the interpretation of our results. Secondly, because clinical renal tissue is extremely difficult to obtain, renal ACE2 concentration and activity could not be determined simultaneously in our study, thus the relationship between renal and uACE2 could not be established. In addition, without a histopathological diagnosis, we could not further investigate the relationship between specific renal injury and uACE2. Thirdly, as using ACEi or ARB could affect the expression of ACE2, we have to exclude the cases with more severe proteinuria and hypertension simultaneously. Consequently, the association between ACE2 and various degrees of proteinuria or hypertension, which may indicate poor progression, cannot be evaluated. Also, the predictive ability of uACE2 to feline renal progression was unevaluable due to the exclusion of these severe cases. Fourthly, the absence of age-matched controls in our study prevents the simultaneous evaluation of the influence of age on both uACE2 concentration and activity. Fifthly, although linear regression and Spearman tests revealed no significant correlation between freeze duration and ACE2 activity (data not shown), it is important to note that we were unable to investigate the potential degradation of activity in the same sample over time. This limitation underscores the need for further exploration in future studies. Finally, in the present study, we did not evaluate other well recognized factors of RAAS, including Ang I, ACE, and Ang II, as well as other emerging RAAS peptides, such as Ang (1–9), Ang (1–7); these could have benefited the interpretation of our results.

In conclusion, it appears that the regulation of uACE2 concentration and activity undergoes alterations in varying directions as renal function declines, particularly in cases with advanced deterioration, among feline individuals afflicted with CKD.

## Data availability statement

The original contributions presented in the study are included in the article/Supplementary material, further inquiries can be directed to the corresponding author.

## Ethics statement

The animal studies were approved by Institutional Animal Care and Use Committee (IACUC) National Taiwan University. The studies were conducted in accordance with the local legislation and institutional requirements. Written informed consent was obtained from the owners for the participation of their animals in this study.

## Author contributions

T-CK: Conceptualization, Data curation, Formal analysis, Investigation, Methodology, Project administration, Software, Validation, Visualization, Writing – original draft, Writing – review & editing. W-LH: Conceptualization, Methodology, Supervision, Validation, Writing – review & editing, Formal analysis. V-CW: Conceptualization, Methodology, Supervision, Writing – review & editing. T-RJ: Methodology, Supervision, Writing – review & editing. P-ST: Methodology, Supervision, Writing – review & editing. Y-JL: Conceptualization, Data curation, Formal analysis, Funding acquisition, Investigation, Methodology, Project administration, Resources, Software, Supervision, Validation, Visualization, Writing – review & editing.
