# Supplementary material for: Urinary angiotensin-converting enzyme 2 and its activity in cats with chronic kidney disease
Source: Front Vet Sci. 2024 May 2;11:1362379. doi: 10.3389/fvets.2024.1362379 (PMC11097973; doi:10.3389/fvets.2024.1362379)
Supplement: Supplementary file 1 [file Table_1.pdf]

Cat Angiotensin I Converting Enzyme 2 (ACE2) ELISA Kit

Catalog #: MBS085876

Lot: 05/2023

PRECISION:

Intra-assay Precision (Precision within an assay): Three samples of known concentration were tested twenty times on one plate to assess intra-assay precision.

Inter-assay Precision (Precision between assays): Three samples of known concentration were tested in six separate assays to assess inter-assay precision.

	Intra-Assay Precision			Inter-Assay Precision		
Sample	1	2	3	1	2	3
n	20	20	20	6	6	6
Mean (ng/ml)	0.24	2.49	6.82	0.26	2.62	7.68
Standard deviation	0.01	0.10	0.34	0.02	0.18	0.31
CV (%)	4.7	4.4	5.0	7.5	6.8	4.0

FOR RESEARCH USE ONLY. NOT FOR USE IN DIAGNOSTIC PROCEDURES.
