# Supplementary figures and images for: Urinary angiotensin-converting enzyme 2 and its activity in cats with chronic kidney disease
Source: Front Vet Sci. 2024 May 2;11:1362379. doi: 10.3389/fvets.2024.1362379 (PMC11097973; doi:10.3389/fvets.2024.1362379)

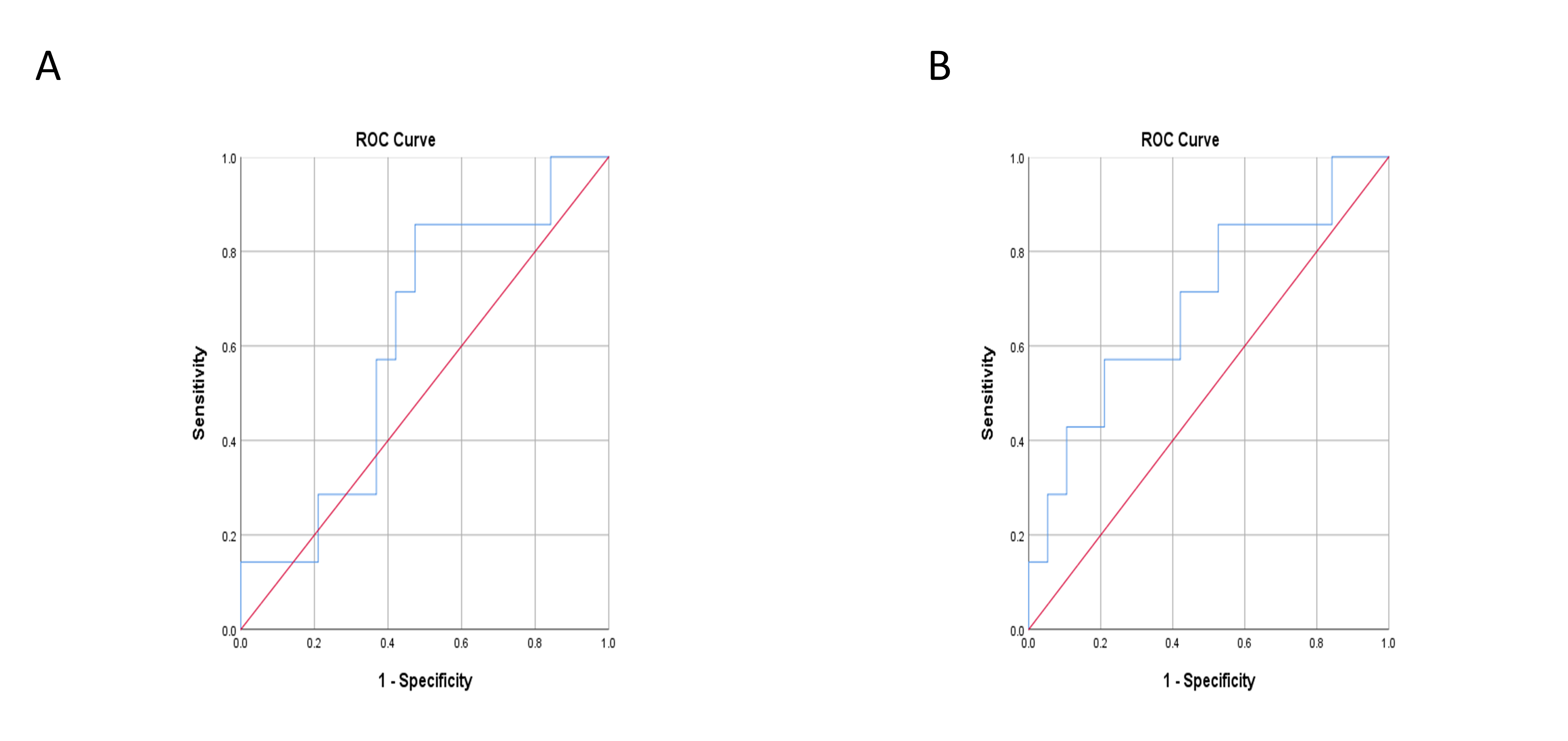

Supplement: Supplementary file 2 [file Image_1.TIF]
